# Novel Use of Valve-in-Valve Transcatheter Mitral Valve Replacement for Severe Bioprosthetic Mitral Valve Stenosis During Pregnancy: A Case Report

**DOI:** 10.1016/j.jscai.2026.104273

**Published:** 2026-03-10

**Authors:** Alexander P. Hoffmann, Yonatan Buber, James M. McCabe

**Affiliations:** a Division of Cardiology, University of Washington, Seattle, Washington; b Division of Cardiology, Beth Israel Deaconess Medical Center, Boston, Massachusetts

**Keywords:** cardio-obstetrics, case report, mitral stenosis, percutaneous valve intervention, structural heart disease, transcatheter mitral valve replacement, valvular heart disease

## Abstract

A 26-year-old woman at 14 weeks gestation with her third pregnancy presented with severe bioprosthetic mitral valve stenosis, which represents very high maternal and fetal risk. Pregnancy termination was recommended but declined, and valve surgery was deemed to be of prohibitive risk. After multidisciplinary discussion, she underwent valve-in-valve transcatheter mitral valve replacement in the second trimester. She had an uncomplicated vaginal delivery at term. This represents a novel use of transcatheter valve replacement to stabilize a pregnant patient with a high–risk valvular lesion. This approach may be considered after multidisciplinary discussion in appropriately selected patients.

## Introduction

Worldwide, rheumatic heart disease, including mitral stenosis (MS), is the most common cardiac condition affecting women of reproductive age. MS caused by bioprosthetic mitral valve (bioMVR) degeneration is more common in the developed world. MS causes heart failure, atrial arrythmias, and pulmonary hypertension, which are poorly tolerated in pregnancy, and maternal/fetal morbidity/mortality is high.^1^ Pregnancy termination and/or valve surgery may be recommended but are often undesired or prohibitive risk.^2^ Valve-in-valve (ViV) transcatheter mitral valve replacement (TMVR) is a novel therapy that can mitigate risks of bioprosthetic MS and valve surgery during pregnancy.^3^

## Case presentation

**Figure 1:**
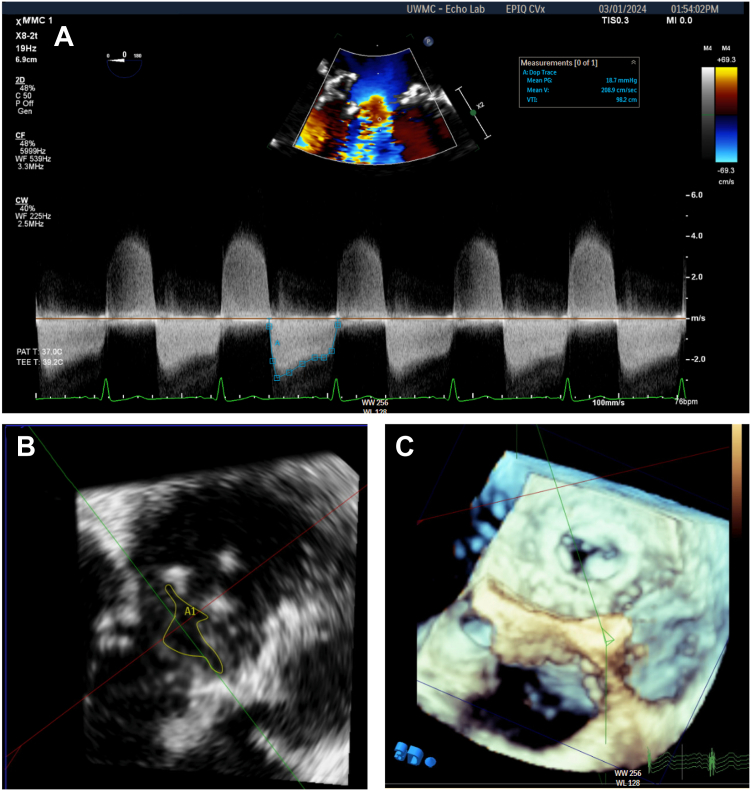
**(A)** Transesophageal echocardiogram before transcatheter mitral valve replacement demonstrating marked inflow acceleration across the bioprosthetic mitral valve (bioMVR). Mean gradient while under anesthesia of 18 mm Hg. (**B**) The 3 dimensional planimetry of the bioMVR. Valve area, 1.2 cm^2^. (**C**) The 3 dimensional image of severely stenotic bioMVR.

A 26-year-old G3P2 woman at 14 weeks’ gestation with a history of remote injection drug use–associated fungal endocarditis status postsurgical bioMVR (25 mm Magna Ease; Edwards Lifesciences) 7 years prior was referred to our institution following an echocardiogram demonstrating severe bioMVR stenosis. The estimated mean inflow gradient was 32 mm Hg, and pulmonary artery systolic pressure (PASP) was 71 mm Hg. The valve area was measured by 3D planimetry from transesophageal echocardiogram (TEE) to be 1.2 cm^2^ (Figure 1B). The mechanism of stenosis was presumed degenerative, and recurrent endocarditis was ruled out. Despite the high gradient and PASP, her symptoms were minimal. A multidisciplinary meeting of maternal and fetal medicine, cardio-obstetrics, cardiac surgery, cardiac anesthesia, and structural cardiology specialists was convened. The consensus was to recommend pregnancy termination, but this was declined by the patient. Valve surgery was considered but declined given high risk of fetal complications during cardiopulmonary bypass. After further discussion, ViV TMVR was offered and performed at 20 weeks’ gestation.

## ViV TMVR

**Figure 2:**
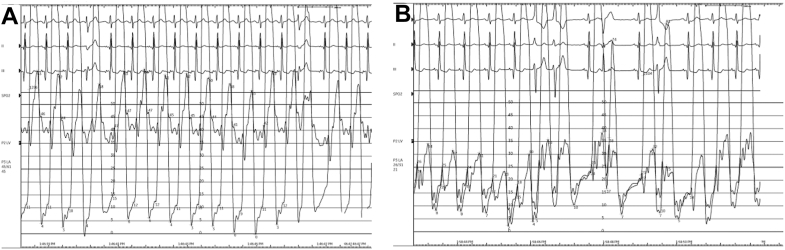
**Intraprocedural simultaneous left atrial and left ventricular pressure tracings.** (**A**) Before transcatheter mitral valve replacement (TMVR), gradient of 28 mm Hg. (**B**) After TMVR, gradient of 3 mm Hg.

Informed consent was obtained from the patient. Under general anesthesia, large-bore femoral venous access was obtained to accommodate a 16F TMVR delivery system. Transseptal puncture was performed with a VersaCross system (Boston Scientific) under TEE guidance. The bioMVR was crossed with a steerable guide and pigtail catheter, and simultaneous left ventricular and left atrial pressures were obtained. Atrial septostomy was performed with a 14-mm balloon inflated to 6 atm. Given the internal dimensions of the surgical prosthesis was 24 mm, valvuloplasty was performed with a 26-mm True balloon to high pressure (Supplemental Video 1) to accommodate a fully expanded 26-mm SAPIEN 3 valve (Edwards Lifesciences), which was deployed under fluoroscopic and TEE guidance (Supplemental Video 2). TMVR position and function was confirmed with TEE, which demonstrated no regurgitation. Repeat left ventricular and left atrial pressures were obtained, which demonstrated marked reduction in mitral inflow gradient (3 mm Hg from 28 mm Hg) (Figure 2A, B). TEE demonstrated trivial left to right shunting through the atrial septal defect, so it was not closed. A total of 148 mGy of radiation and no contrast were used. Estimated PASP was reduced to 26 mm Hg on postprocedural echocardiogram. Low-dose aspirin was initiated. She proceeded to have an uncomplicated pregnancy with vaginal delivery at term and an unremarkable postpartum period.

## Discussion

MS due to bioMVR degeneration, particularly with pulmonary hypertension, is a high–risk valvular lesion that may be encountered in women of child-bearing age or during pregnancy. Guidelines recommend avoidance of pregnancy in this context, and pregnancy termination may be recommended.^4^ In native valve rheumatic heart disease–related MS, percutaneous mitral valve commissurotomy can be performed in select patients during pregnancy, but this is not performed in cases of bioMVR stenosis.^5^ In patients with severe bioMVR stenosis who do not elect for pregnancy termination, maternal risk for major cardiac events during pregnancy and into the post-partum period is high, possibly as much as 50%, driven predominantly by heart failure.^1^ Risk of fetal complications is also high, including intrauterine growth restriction, premature birth, and stillbirth.^6^ In the event of severe maternal decompensation, valve surgery during pregnancy may be recommended but carries high risk of fetal complications, including fetal demise.^7^

ViV TMVR is an emerging alternative to redo surgical valve replacement in patients with failing bioMVRs. A recent meta-analysis demonstrated ViV TMVR had high technical success (94.8%), with shorter hospital length of stay (7.2 vs 10.3 days) and lower risk of short-term morbidity and mortality compared to redo surgical MVR (6.4% vs 8.4% mortality, 1.9% vs 5.5% stroke risk, 6.7% vs 10.6% risk for major bleeding, and 3.0% vs 12.5% risk for pacemaker implantation). Valve embolization occurred in 1.8% of cases.^8^ While not randomized and therefore not accounting for selection bias, there is growing evidence that supports ViV TMVR as a feasible and relatively low risk alternative to surgery in this high-risk population. A single report of successful ViV TMVR performed during pregnancy exists in the literature,^9^ and a small series of women contemplating pregnancy with high–risk mitral lesions suggests that ViV TMVR before pregnancy may ameliorate risk of adverse outcomes during pregnancy.^3^

ViV TMVR was used in this case to mitigate risks associated with severe bioMVR stenosis and pulmonary hypertension during pregnancy. In our case, TMVR was performed in the second trimester when most fetal organogenesis is complete, the fetal thyroid is inactive, and a relatively small uterus minimizes distance from the chest to reduce radiation exposure to the fetus. The procedural approach in this case was similar to the reported ViV TMVR case series.^10^ Following TMVR, an immediate and marked reduction in the invasively measured diastolic mitral inflow gradient was observed (28 mm Hg reduced to 3 mm Hg) (Figure 2A, B). While the optimal anticoagulation/antiplatelet strategy following TMVR is not known, anticoagulation is frequently initiated. Given the unique risks of thrombosis/bleeding during pregnancy in this case, aspirin monotherapy was initiated and planned to be continued following delivery. The remainder of the pregnancy was well tolerated, and follow-up echocardiograms in the second and third trimesters showed stable valve gradients and PASP. The patient had an uncomplicated vaginal delivery at term, followed by an uneventful postpartum period. Method of delivery should follow standard obstetric indications with a normally functioning bioprosthetic valve. Cesarean delivery is associated with longer hospitalizations and higher bleeding/infectious risk without improved maternal cardiac outcomes. Counseling regarding future pregnancies following ViV TMVR should be individualized and guided by postprocedural valve performance and longitudinal echocardiographic assessment. Future pregnancies should be followed in a dedicated cardio-obstetrics clinic.

## Conclusions

ViV TMVR is an emerging alternative to valve surgery during pregnancy in women presenting with severe bioMVR stenosis. A patient-centered approach by a multidisciplinary team is critical, and this procedure should be considered at high volume comprehensive valve centers in the hands of experienced operators. Long-term outcomes of ViV TMVR, including in pregnant patients, are not yet known.

## Declaration of competing interest

James M. McCabe is a consultant for Edwards Lifesciences. Alexander Hoffmann and Yonatan Buber have nothing to disclose.

## Footnotes

To access the supplementary material accompanying this article, visit the online version of the *Journal of the Society for Cardiovascular Angiography & Interventions* at 10.1016/j.jscai.2026.104273.
